# Analysis of State-Specific Differences in Childhood Vaccination Coverage in Rural India

**DOI:** 10.3390/vaccines7010024

**Published:** 2019-02-24

**Authors:** Nijika Shrivastwa, Abram L. Wagner, Matthew L. Boulton

**Affiliations:** 1 Department of Epidemiology, University of Michigan, Ann Arbor, MI 48109, USA; nijikas@umich.edu (N.S.); mboulton@umich.edu (M.L.B.); 2 Department of Internal Medicine, Division of Infectious Disease, Michigan Medicine, University of Michigan, Ann Arbor, MI 48109, USA

**Keywords:** India, vaccination coverage, religion

## Abstract

There is little research on state-level differences in child health outcomes in India. The aim of this study was to identify state-level characteristics that relate to childhood immunizations. Most state-level characteristics came from the 2011 Indian Census. Individual-level data and other state-level characteristics were obtained from the 2007–2008 District Level Household and Facility Survey. Predictors of full vaccination were assessed with logistic regression models. Among 86,882 children 12–36 months, 53.2% were fully vaccinated. Children living in bigger households (≥7 members), born in non-institutional settings, and female had lower odds of complete vaccination. Individuals living in states in the mid-range of poverty had lower odds of full vaccination compared to those in lower or higher poverty states (3rd vs. 1st quintile: odds ratio [OR]: 0.36, 95% confidence interval [CI]: 0.30, 0.42). Greater average population per primary health center was associated with decreased odds of full vaccination (5th vs. 1st quintile: OR: 0.37, 95% CI: 0.30, 0.47). Vaccination coverage in India can be explained by a complex interplay of individual- and state-level factors. Solutions to increasing vaccination must be multisectoral and acknowledge the cultural and socio-economic diversity that influences an individual child’s vaccination coverage along with within-state disparities.

## 1. Introduction

India experiences some of the highest preventable childhood mortality of any country in the world with over 600,000 deaths annually in children aged 1 to 59 months from vaccine-preventable diseases [1]. Among the 19.5 million children worldwide in 2016 who did not receive the third dose of diphtheria-pertussis-tetanus vaccine (DPT3), a commonly accepted proxy for the effectiveness of a country’s immunization program, 16% were from India [2]. 

Chronically low vaccination coverage combined with pronounced inequities in immunization receipt across socio-economic and demographic groups continues to exist in India [3], despite the existence of a long-standing national immunization program that provides recommended vaccines free of charge to all infants [4]. India’s national District Level Household and Facility Survey in 2008 (DLHS3) estimated the percentage of fully-vaccinated children 12–23 months of age as 54%, although this masks the extreme variation in vaccination coverage across states, with coverage ranging from 13% in Arunachal Pradesh to 82% in Tamil Nadu. 

Given the wide variation in vaccination coverage across Indian states, its seems highly plausible that the lack of progress, at least in part, in improving vaccination coverage and vaccination inequities in India is due to state-specific factors [5]. Numerous studies have focused on individual predictive factors for vaccination in India, including gender, age, and birth order. Other studies have focused on household factors, such as family size, number of children below 3 years old, household wealth, and maternal education [3,5,6,7,8,9]. 

India is a uniquely diverse country with over 2000 spoken dialects and languages, reflecting a tremendous variation in local and regional traditions, cultural practices, religious beliefs, and socio-economic pressures. Geographic clustering of certain groups does occur within states or regions. With this degree of heterogeneity, state-level differences could be expected to exert an influence on the expression of individual-level predictors of childhood vaccination, especially since many state-level policies and programs directly impact health care availability and accessibility, including immunization services. The composition of individuals belonging to specific religions and castes can be considered proxy indicators for cultural diversity of a state.

Religion and caste reflect cultural designations that influence parental beliefs and attitudes toward health-seeking behaviors including vaccination decisions about their children [5,9]. There are four major religions in India: the Hindu majority as well as Muslims, Christians, and Sikhs. Other groups, such as Buddhists, Jains, Jews, and Parsis, individually make up less than one percent of the total population. A previous analysis of DLHS3, which accounted for state-level differences, showed that compared to the majority Hindus, Sikh children had better and Muslim children worse vaccination outcomes [3]. Individuals in scheduled castes (SC) and scheduled tribes (ST) are subject to social discrimination and are historically less privileged than other backward castes (OBC), and “others,” who represent the most highly privileged caste grouping. Low caste status can impact the ability to access governmental services, including vaccination [10,11,12,13,14]. However, previous studies have not taken into account the impact of state-level factors (like proportion from different religious groups or from SC/ST) on vaccination.

There is a dearth of research investigating state-level differences in risk factors for child health outcomes in India. A 2010 study reported variation in disparities for a number of child health outcomes by gender and residence in different Indian states [15]. Another study by De and Bhattacharya examined the factors affecting childhood vaccination in the Indian states of Madhya Pradesh, Bihar, Uttar Pradesh, and Rajasthan, and reported lower vaccination coverage among Muslim children compared to children from other religious groups in all states except Madhya Pradesh, where vaccination coverage among Muslim children was better than all other religious groups [16]. A 2008 study comparing childhood immunizations in Maharashtra and Bihar reported that the probability of complete vaccination coverage was higher for children residing in rural areas compared to urban areas of Maharashtra, which differed from Bihar where higher vaccination coverage was observed in the urban areas [17]. In an another study by Kumar et al., greater overall inequity in vaccination coverage was observed in Maharashtra compared to Bihar, an economically much poorer state [18]. 

Although these studies show state-specific differences between various risk factors and child health outcomes, including vaccination, all were characterized by significant limitations. The majority compared relatively few states and most are largely descriptive in nature [19]. Additionally, these studies [16,18] often use overly broad and sometimes inconsistent predictor categories. For example, studies often categorize religion into Hindu and non-Hindu ignoring the rich diversity in religious affiliation among non-Hindus; dichotomize caste into SC/ST and non-SC/ST; and simplify household wealth. 

Contextual state-level variables include characteristics of the communities in which children reside, including social and economic variables, as well as the availability of healthcare resources. Healthcare services accessibility and availability is an important predictor for vaccination [5]. Such community-level factors may influence parental decisions for the receipt of preventive services (such as vaccination for their children) independent of individual-level characteristics [20]. For example, an uneducated mother living in a politically progressive state that has better vaccination outreach programs may be more likely to receive vaccination for her children because of her social interactions, accessibility to healthcare services, and prevailing social norms than an educated mother who resides in a less progressive or poorer Indian state. Thus, a clearer understanding of the relative influence of state-level factors in the context of important individual factors like religion and caste is needed in order to shape interventions aimed at improving delivery of childhood vaccinations at the population level. This understanding is particularly important because the provision of health care services in India varies widely across states, programs are implemented differently, and states vary in their status relative to the epidemiological transition [21].

Little is known about the effect of intra-country regional differences on individual immunization coverage, especially in a low- or middle-income country like India, since most studies on this topic have taken place in high-income countries. For example, a survey in the Netherlands in 2009 found community political ideologies and socioeconomic status to be associated with human papillomavirus vaccination [22]. A study of Hawaiian kindergartners in 2002–2003 found that Census tract-level statistics, including education, urbanicity, and ethnic proportions, were significantly associated with up-to-date immunizations [23]. And one measure of vaccination coverage, the occurrence of non-medical vaccination waivers, has been linked to U.S. state-level policies about waivers [24].

This study examines state-specific differences in childhood vaccination coverage among the rural population in India, using a nationally representative sample of children aged 12–36 months. It also presents analysis of state-specific socio-cultural factor rates that are known at the individual level to be predictors of childhood vaccination in India. The specific objectives of this study are: (1) to analyze state-specific differences in the association of religion with childhood vaccination status, and (2) to identify the state-level characteristics that are predictive of childhood immunizations.

## 2. Materials and Methods

### 2.1. Data Source and Sample Design

Study data were derived from two datasets. Individual-level data and a few state-level characteristics were obtained from DLHS3 collected from 720,320 households in 26 Indian states (out of 28 states at the time) during December 2007 to December 2008. The target population included children born from January 2004 to December 2007 who were in the age range of 12–36 months at the time of the interview. In cases where more than one child in a household met these criteria, the most recent born child in the family was selected in order to reduce the minor effects of two children and to eliminate the need for multilevel models in the analysis. The record for each child includes selected characteristics of the child, mother, household, and state information. Most information in the DLHS3 dataset was collected through in-person interviews with trained interviewers, although interviewers used vaccination cards to record vaccination dates if these cards were available (otherwise maternal recall was used). The DLHS3 dataset was chosen so that future studies can analyze trends in coverage over time and because data were collected shortly after the start of the National Rural Health Mission in 2005, whose mission was to increase access to health care in rural areas of India [25]. The analysis in this manuscript only includes individuals from rural areas of India. The DLHS3 dataset is available from: http://rchiips.org/PRCH-3.html.

Most state-level data came from the 2011 Indian Census, which is one of the only nationally representative surveys in India to collect detailed information on socioeconomic status, including religious affiliation. Over 2.7 million Census officials visited households in over 600,000 villages. The 2011 Census can be found at: https://data.gov.in/resources/primary-census-abstract-2011-india.

### 2.2. Outcome Variable

The vaccination status of the child was coded as either fully-vaccinated or not fully-vaccinated. Full vaccination was one dose bacillus Calmette-Guerin (BCG, tuberculosis), three doses DPT; and one dose measles-containing vaccine (MCV). Children receiving fewer doses of any of these vaccines than the full complement or had not received doses of any of these vaccines were considered to be not fully-vaccinated. 

### 2.3. State-Level Factors

State-level variables considered for the analysis were healthcare services availability and socio-cultural and socio-demographic characteristics. Using data from DLHS3, we estimated average population per Primary Health Center (PHC)—an indicator of the availability and accessibility of immunization services, and the percentage of poor households (i.e., in the lowest national wealth quintile). From the 2011 Census state-level data, we obtained the percent Muslim population within the state and population density. States were ranked on these characteristics, and categorized into quintiles.

### 2.4. Individual-Level Factors

Factors related to the child and to the mother were obtained from DLHS3 and included child’s gender and age, mother’s age at child’s birth, religion, caste, maternal education, maternal receipt of antenatal care (ANC) services, and place of delivery. 

### 2.5. Statistical Analysis

All descriptive and analytical analyses used appropriate survey procedures. Survey weights were used to enable unbiased estimation of population characteristics. Analyses used strata (i.e., rural vs. urban) and cluster variables (i.e., the primary sampling unit), which accounted for the complex design characteristics, and which allowed for the analyses to output valid standard errors. The Taylor series linearization method was used to calculate variance of the parameter estimates. Descriptive statistics for the individual- and state-level characteristics were examined before proceeding to regression modeling. Two logistic regression models were constructed. The first only included individual-level predictors, and the second included state-level socio-cultural and socio-demographic characteristics. All predictors were selected a priori based on past literature.

### 2.6. Ethical Approval

The team that collected data adhered to international ethical norms and the Helsinki Declaration, including receiving an ethical review of the study at the International Institute for Population Sciences in Mumbai, India, and obtaining informed consent prior to enrolling individuals into the study. Participants were notified that participation was voluntary, that they could withdraw at any time, and that responses would be confidential. Participants were also given contact information of personnel at the International Institute for Population Sciences if they had questions about the ethics or research content. This manuscript was limited to a secondary analysis of this dataset which contained no personally identifiable information. The University of Michigan Health Sciences and Behavioral Sciences Institutional Review Board reviewed our protocol and deemed the secondary data analysis exempt from ethical review from the University of Michigan (HUM00077627).

## 3. Results

The unweighted sample size of children residing in rural areas and who were 12–36 months eligible was 86,882. Table 1 shows the distributions of socio-economic characteristics and vaccination status of the study population. A total of 53.2% of the target population was fully vaccinated. The majority was Hindu (78.3%), and Muslims (13.0%) were the largest minority. Approximately half (50.3%) had mothers had no formal schooling, and most births (65.2%) were non-institutional. Many mothers (33.3%) had not received any ANC services.

Table 2 shows characteristics of the 26 Indian states that were included in the analysis. The mean percentage of population living in the lowest wealth quintile was 15.8% (Range: 0.5% to 48.5%). The mean state population density ranged from 17 to 1102 persons per square kilometer. The mean proportion Muslim by state was 11.9%, ranging from 1.1% to 67.0%.

**Figure 1:**
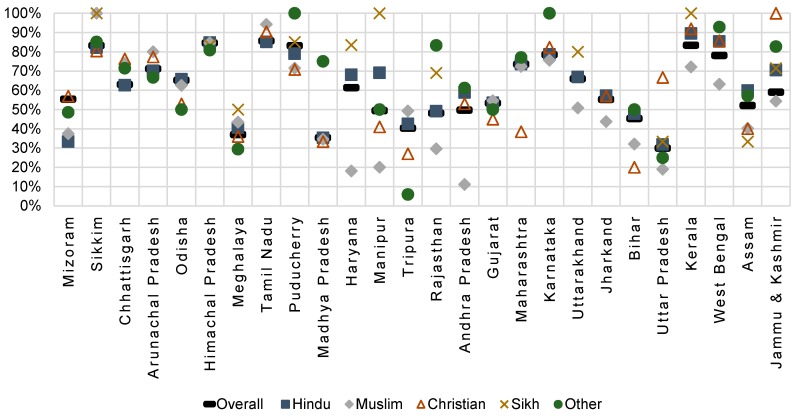
Percentage of fully-vaccinated children aged 12 to 36 months by religion and state of residence, DLHS3. States are ordered from lowest proportion Muslim to highest proportion Muslim.

Figure 1 shows state variation in the percentage of fully vaccinated children overall and among different religious groups. Substantial inter-state variation was observed, and lowest full vaccination coverage was in Uttar Pradesh (30.0%) and Madhya Pradesh (35.4%). In other states like Kerala, Tamil Nadu, Punjab, and Sikkim, full vaccination coverage was above 80%. Muslim children had the lowest percentage of fully-vaccinated children in multiple states including Jammu & Kashmir, Uttar Pradesh, Uttarakhand, Rajasthan, Manipur, Haryana, Karnataka, and Kerala. However, in the states of Tamil Nadu, Arunachal Pradesh, Tripura, and Sikkim, Muslim children had the highest rates full-vaccination coverage. Sikh children had some of the highest levels of full-vaccination in every state, except in Assam. 

Using as an indication of high disparity a difference of 10% or more in full vaccination coverage among different religious groups within a state, the high disparity states were Jammu & Kashmir, Haryana, Rajasthan, Uttar Pradesh, Manipur, Arunachal Pradesh, Tripura, and Kerala. The states of Gujarat and Himachal Pradesh had little disparity.

Table 3 displays predictor odds ratios from two different models—one only including individual-level characteristics, and the other including individual- and state-level characteristics. We found that children living in bigger households (≥7 members), born in non-institutional settings, and female had lower odds of complete vaccination. The strength of associations did not substantially change after adjusting for state-level factors, although adjusting for state-level factors slightly attenuated the strength of association related to maternal education and number of ANC visits. Individuals living in states with more individuals in the lowest wealth quintile had significantly decreased odds of full vaccination (5th vs. 1st quintile: OR: 0.76, 95% CI: 0.66, 0.88). Greater average population per PHC was also associated with decreased odds of full vaccination (5th vs. 1st quintile: OR: 0.37, 95% CI: 0.30, 0.47).

## 4. Discussion

This study investigated the association of individual-and state-level factors with vaccination status of children in India using a nationally representative dataset. Echoing past research [26,27], children living in bigger households, born in non-institutional settings, and female had lower odds of complete vaccination. Little was previously known about how state-level characteristics impact vaccination coverage. We found that individuals had lowest full vaccination coverage in states in the middle range of poverty, whereas wealthier and poorer states had higher vaccination coverage, and states which had greater population per primary health center had lower odds of full vaccination coverage.

States with better immunization services and higher full vaccination coverage benefit all residents of the state thereby reducing disparities. In those states with the lowest overall full-vaccination coverage, all religious groups fared equally poorly. In contrast, states with highest disparities were those with mid-level overall full vaccination coverage ranging from 45% to 63%. It is likely that when the healthcare resources such as immunization services are neither very low nor very high, everyone either benefits or suffers alike. Further, when full vaccination coverage is intermediate, certain groups preferentially receive more services or greater access to services resulting in greater disparities in full-vaccination coverage across religions. 

This difference by religion was apparent within states and across states. Living in a state with poor availability of primary health care services increases the risk for incomplete childhood vaccination, likely because vaccinations are commonly delivered by these clinics. Similarly, states with higher population densities had lower full-vaccination which may result from greater population pressure on immunization services and related resources. 

The impact of maternal education or ANC visits on vaccination status slightly diminished after controlling for state-level characteristics. Similar results have been reported in previous studies from India and Bangladesh, with the strength of maternal education declining substantially after controlling for community-level socio-economic status [28,29,30]. Vikram et al., demonstrated that well-educated mothers tend to live in villages with other well-educated mothers and had better access to medical care [28]. Based on these findings, when there is a higher concentration of illiterate people in the areas with poorer healthcare services, then improving access to PHCs could help address inequities in vaccination coverage in areas characterized by lower levels of maternal education. This is especially important since half of Indian mothers in the survey lacked any formal schooling. Maternal education has long been established as an important predictor of childhood vaccination in every country, including India, but in a nation with such a disproportionately large number of mothers who lack formal schooling, educational improvements is a long-term, not short-term, solution. Providing more accessible immunization services through the already existing national network of PHCs might be an easier and more rapid way to address this discrepancy in immunization uptake.

Important individual level characteristics that did not appear to be influenced by state-level factors included household size, gender, and birth location (institutional vs. not). Several studies have pointed to the existence of gender disparities in accessing immunization services in India [8]. This may indicate that making healthcare services more readily available may not be sufficient to address this issue. Rather, there may be a need to implement targeted intervention programs in some states or regions of India to specifically decrease gender disparities in access to care. Similarly, births in non-institutional setting are often an indication of the non-availability or non-utilization of primary health care, although non-institutional birth can also be due to cultural practices and beliefs. For example, a key reason for poor uptake of reproductive child health services by women in India is the lack of perceived needs to use medical care [31]. Finally, children living in bigger households (seven or more members) were less likely to be fully-vaccinated. This may well be due to the obvious factor that the mothers living in large families are busier and have less time for individual child care.

State-level poverty is treated here as a proxy for the presence of progressive state policies. There is a “V”-shaped relationship between state-level poverty and vaccination coverage rate, in that the states with the highest and least degrees of poverty had higher vaccination coverage than states of average wealth. It could be that the states with higher proportions of poor people have recognized the need for implementing special outreach programs for the poor and/or receive more governmental assistance for these programs. For example, India’s National Rural Health Mission was established in 2005 with the goal of reducing maternal mortality, infant mortality, and the fertility rate in rural, remote regions through the promotion of basic medical services, like institutional delivery, neonatal care, and immunizations [25]. Therefore, certain states could have higher coverage despite a higher proportion of poor populations. Large inequities in the full-vaccination coverage between the rich and poor populations may be due to a higher overall vaccination rate within a state. Certain states, such as Maharashtra, Rajasthan, Uttar Pradesh, Odisha, Manipur, and Bihar, have had large differences between the richest and poorest strata according to a 2013 study on vaccination inequity [19].

As average population served by PHC increases, there is a decrease in vaccination coverage rates. According to the Indian Public Health guidelines, the average population served by each PHC should be approximately 30,000 and when the number of people served by a PHC exceeds that, it may negatively impact service delivery, including vaccinations. The direct implication of this finding is that increasing the number of PHCs could help limit the over-burdening of existing health centers. A review study [5] showed that closer proximity to a health center was positively associated with a child’s full vaccination status, a finding mirrored here. Additional PHCs that are appropriately distributed would create a greater probability of placing children closer to a PHC and giving them greater access to preventive services. 

### Strengths and Limitations

This is the first study that investigates state-specific differences in childhood vaccination status in India while also describing differences in vaccination coverage rates of different religious groups across states. One limitation is the age of the data, although we note that there were very few changes in vaccination coverage between 2007 and 2012 (for instance, measles vaccination coverage was 84.1% in DLHS3 and 82.6% in DLHS4—a dataset from 2012–2013 [32]), and vaccination coverage in India has likely remained stagnant over the past decade.

The sample is restricted to the rural population only which enabled us to examine various variables that were only collected in rural areas in the DLHS3 dataset. Since 80% of the DLHS3 data pertain rural residents, there was a sufficiently large sample size to permit investigation of various associations with sufficient statistical power in most states. However, given the general disparities between urban and rural areas in India, the results of this study may not be generalizable to urban populations. This study showed state-specific factors associated with childhood vaccinations and state specific factors that may influence the individual level predictors of vaccinations, especially for a number of states. The study is also unique in its examination of the modification of religion’s impact on vaccination status by size of Muslim population, a proxy for cultural diversity. Future studies could also include other measures of socio-economic diversity, including the proportion of individuals who are SC or ST within the state. 

State-level variables were categorized into quintiles. The overall impact of one state-level predictor in a quintile can be dominated by a larger state in that quintile. Given the complex interactions between state-level characteristics and the health policy environment, modelling such factors is challenging. This study represents a first step towards understanding the impact of the combination of state and individual factors on vaccination completion. Future studies investigating the influence of policy and cultural factors on individual vaccination status should examine district level factors and their influence, as district level factors have more proximal association with vaccination status. Future studies could also examine the impact of more finely grained policy initiatives in different states (for instance, roll out of Mission Indradhanush or the quality of ASHAs) differentially impacts vaccination uptake and the provision of other maternal and child health care services.

## 5. Conclusions

India has one of the largest populations of unvaccinated and under vaccinated children in the world, and increasing the proportion of children who have received the full set of immunizations would go a long way in reducing childhood morbidity and mortality. We found that full vaccination coverage within the country could be explained by a complex interplay of individual- and state-level factors. On an individual level, children living in bigger households, born in non-institutional settings, and female had lower odds of complete vaccination. At a state level, individuals had lowest full vaccination coverage in the mid-range of poverty levels by state, whereas wealthier and poorer states had higher vaccination coverage. Greater average population per PHC was also associated with decreased odds of full vaccination. Solutions to increasing vaccination coverage in India must be multisectoral and acknowledge the cultural and socio-economic diversity that influences an individual child’s vaccination coverage as well as the disparity in vaccination within the state.

## Figures and Tables

**Table 1:** Individual-level characteristics of Indian children aged 12 to 36 months, DLHS3.

Characteristic	Category	Un-Weighted Count	Weighted % (95% CI)
Vaccination status		86,882	
	Fully vaccinated		53.2 (52.7, 53.7)
	Not fully vaccinated		46.8 (46.3, 47.3)
Religion		85,459	
	Hindu		78.3 (77.9, 78.7)
	Muslim		13 (12.5, 13.5)
	Christian		5.3 (5.1, 5.6)
	Sikh		2.0 (1.9, 2.2)
	Other		1.4 (1.3, 1.5)
Caste		85,221	
	Scheduled caste		19.6 (18.8, 20.6)
	Scheduled tribe		20.4 (20.0, 20.9)
	Other backward caste		40.2 (39.4, 41.0)
	Other		19.8 (19.4, 20.2)
Wealth quintile		86,872	
	Poorest		24.8 (24.3, 25.3)
	Poor		24.8 (24.4, 25.2)
	Middle		22.5 (22.1, 22.8)
	Rich		18.5 (18.1, 18.8)
	Richest		9.5 (9.1, 9.8)
Household size		86,882	
	3 members		7.6 (7.4, 7.8)
	4-5 members		30.4 (30.1, 30.7)
	6-7 members		29.1 (28.7, 29.5)
	≥8 members		32.9 (32.6, 33.2)
Maternal Age		86,882	
	≤18 years		8.3 (8.1, 8.5)
	19-25 years		53.4 (52.8, 53.9)
	26-35 years		33.6 (33.1, 34.1)
	≥36 years		4.7 (4.6, 4.9)
Child’s gender		86,879	
	Male		52.7 (52.4, 53.0)
	Female		47.3 (47, 47.7)
Maternal education		86,882	
	No formal schooling		50.3 (49.6, 50.9)
	1-6 years		18.2 (17.9, 18.4)
	≥7 years		31.5 (30.9, 32.2)
Delivery location		85,189	
	Government institution		20.7 (20.4, 21.0)
	Private institution		14.1 (13.7, 14.5)
	Non-institutional		65.2 (64.6, 65.8)
Antenatal care		86,882	
	No visits		33.3 (32.8, 33.9)
	1-2 visits		41.9 (41.4, 42.5)
	3-6 visits		19.9 (19.5, 20.3)
	≥7 visits		4.9 (4.7, 5.0)
Maternal tetanus vaccination		85,192	
	No		32.7 (32.2, 33.2)
	Yes		67.3 (66.8, 67.8)

**Table 2:** Indian state-level characteristics from DLHS3 and the 2011 Census.

Variable	Number of States	Mean	SD	Minimum	Maximum
Scheduled caste (%)	25	14.2	7.9	0.1	31.9
Scheduled tribe (%)	24	22.7	26.0	0.6	94.4
Average population per PHC	26	42,138	36,479	5216	158,275
Population density (population/km^2^)	26	373	297	17	1102
Muslim (%)	26	11.9	13.8	1.1	67.0
In lowest wealth quintile (%)	26	15.8	14.1	0.5	48.5

Notes: PHC, primary health center; SD, standard deviation.

**Table 3:** Adjusted odds ratios (AOC) and confidence intervals (CI) for full-vaccination of children aged 12 to 36 months.

Characteristic	Category	Individual-Level Predictors AOR (95% CI)	Individual- and State-Level Predictors AOR (95% CI)
**Religion**	Hindu	reference	reference
	Muslim	0.55 (0.52, 0.59)	0.56 (0.52, 0.60)
	Christian	0.70 (0.64, 0.77)	0.77 (0.69, 0.87)
	Sikh	2.30 (1.96, 2.69)	1.21 (0.99, 1.49)
	Other	1.43 (1.22, 1.67)	1.10 (0.93, 1.31)
**Caste**	Other	reference	reference
	Other backward caste	0.81 (0.77, 0.86)	0.87 (0.82, 0.92)
	Scheduled caste	0.89 (0.83, 0.94)	0.89 (0.83, 0.95)
	Scheduled tribe	0.84 (0.79, 0.89)	0.78 (0.73, 0.84)
**Wealth quintile**	Poorest	reference	reference
	Poor	1.19 (1.12, 1.26)	1.17 (1.11, 1.24)
	Middle	1.30 (1.23, 1.37)	1.32 (1.25, 1.39)
	Rich	1.48 (1.38, 1.58)	1.52 (1.41, 1.64)
	Richest	1.57 (1.44, 1.70)	1.76 (1.60, 1.92)
**Household size**	3 members	reference	reference
	4–5 members	0.99 (0.93, 1.05)	0.95 (0.90, 1.01)
	6–7 members	0.94 (0.88, 1.01)	0.92 (0.86, 0.99)
	7+ members	0.81 (0.76, 0.86)	0.81 (0.76, 0.87)
**Maternal age**	≤18 years	0.96 (0.92, 1.01)	0.93 (0.88, 0.98)
	19–25 years	reference	reference
	26–35 years	1.00 (0.97, 1.04)	1.04 (1.01, 1.08)
	≥36 years	0.92 (0.84, 0.99)	0.96 (0.89, 1.04)
**Child’s gender**	Female vs. male	0.92 (0.89, 0.95)	0.91 (0.88, 0.94)
**Child’s age**	Months (continuous)	1.00 (1.00, 1.01)	1.00 (1.00, 1.01)
**Maternal education**	No formal School	reference	reference
	1–6 years	1.45 (1.39, 1.52)	1.35 (1.29, 1.42)
	≥7 years	1.91 (1.83, 1.99)	1.75 (1.67, 1.84)
**Delivery location**	Government institution	reference	reference
	Private institution	0.91 (0.86, 0.96)	0.92 (0.87, 0.98)
	Non-institutional	0.72 (0.69, 0.75)	0.76 (0.73, 0.79)
**Antenatal care**	No visits	reference	reference
	1–2 visits	1.10 (0.98, 1.22)	1.15 (1.02, 1.28)
	3–6 visits	1.96 (1.74, 2.20)	1.57 (1.37, 1.79)
	≥7 visits	2.28 (2.01, 2.58)	1.52 (1.33, 1.74)
**Maternal TT shot**	Yes vs. no	2.18 (1.96, 2.41)	2.14 (1.92, 2.38)
**In lowest wealth quintile**	1st quintile		reference
	2nd quintile		0.62 (0.54, 0.71)
	3rd quintile		0.36 (0.30, 0.42)
	4th quintile		0.56 (0.49, 0.64)
	5th quintile		0.76 (0.66, 0.88)
**Average population per Primary Health Center**	1st quintile		reference
	2nd quintile		1.08 (0.90, 1.28)
	3rd quintile		1.35 (1.15, 1.59)
	4th quintile		0.67 (0.56, 0.80)
	5th quintile		0.37 (0.30, 0.47)
**Population density**	1st quintile		reference
	2nd quintile		1.08 (0.94, 1.23)
	3rd quintile		3.07 (2.64, 3.58)
	4th quintile		1.11 (0.99, 1.25)
	5th quintile		1.29 (1.15, 1.46)
**Proportion Muslim**	1st quintile		reference
	2nd quintile		0.98 (0.83, 1.16)
	3rd quintile		0.72 (0.63, 0.83)
	4th quintile		1.70 (1.47, 1.97)
	5th quintile		0.88 (0.75, 1.02)
